# *N*-(4-Bromo­benzyl­idene)naphthalen-1-amine

**DOI:** 10.1107/S1600536812019800

**Published:** 2012-05-12

**Authors:** Ruitao Zhu, Yuehong Ren, Yuewen Zhang

**Affiliations:** a Department of Chemistry, Taiyuan Normal University, Taiyuan 030031, People’s Republic of China

## Abstract

The title mol­ecule, C_17_H_12_BrN, is in a *E* conformation with respect to the C=N bond. The dihedral angle between the naphthalene ring system and the benzene ring is 53.26 (3)°.

## Related literature

For general background on the properties of Schiff bases, see: Chen *et al.* (2008 ▶); May *et al.* (2004 ▶); Weber *et al.* (2007 ▶). For related structures, see: Zhu *et al.* (2010 ▶); Harada *et al.* (2004 ▶); Tariq *et al.* (2010 ▶).
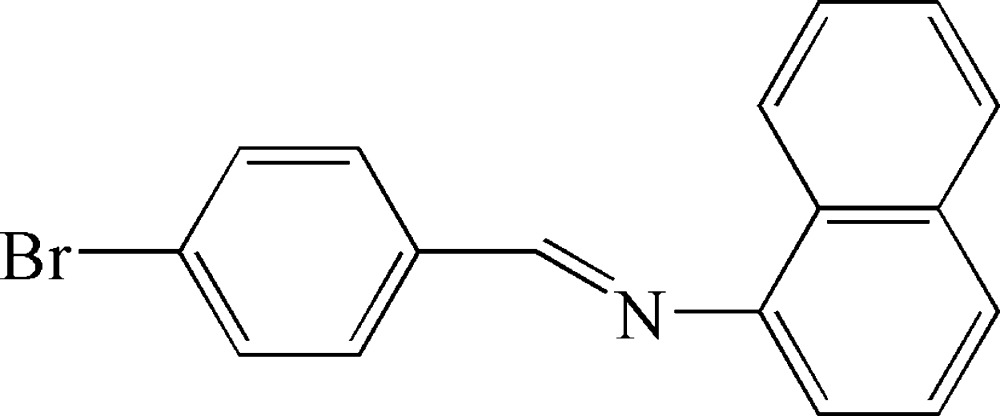


## Experimental

### 

#### Crystal data

C_17_H_12_BrN
*M* *_r_* = 310.19
Monoclinic, 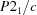

*a* = 7.0823 (6) Å
*b* = 25.555 (2) Å
*c* = 7.5712 (5) Å
β = 94.431 (1)°
*V* = 1366.19 (18) Å^3^
*Z* = 4
Mo *K*α radiation
μ = 2.99 mm^−1^
*T* = 298 K
0.45 × 0.41 × 0.28 mm

#### Data collection

Bruker SMART CCD diffractometer
Absorption correction: multi-scan (*SADABS*; Sheldrick, 1996 ▶) *T*_min_ = 0.346, *T*_max_ = 0.488
6750 measured reflections
2405 independent reflections
1643 reflections with *I* > 2σ(*I*)
*R*_int_ = 0.080

#### Refinement

*R*[*F* ^2^ > 2σ(*F* ^2^)] = 0.043
*wR*(*F* ^2^) = 0.100
*S* = 1.03
2405 reflections
173 parameters
H-atom parameters constrained
Δρ_max_ = 0.45 e Å^−3^
Δρ_min_ = −0.32 e Å^−3^

### 

Data collection: *SMART* (Bruker, 2007 ▶); cell refinement: *SAINT* (Bruker, 2007 ▶); data reduction: *SAINT*; program(s) used to solve structure: *SHELXS97* (Sheldrick, 2008 ▶); program(s) used to refine structure: *SHELXL97* (Sheldrick, 2008 ▶); molecular graphics: *SHELXTL* (Sheldrick, 2008 ▶); software used to prepare material for publication: *SHELXTL*.

## Supplementary Material

Crystal structure: contains datablock(s) I, global. DOI: 10.1107/S1600536812019800/lh5468sup1.cif

Structure factors: contains datablock(s) I. DOI: 10.1107/S1600536812019800/lh5468Isup2.hkl

Supplementary material file. DOI: 10.1107/S1600536812019800/lh5468Isup3.cml

Additional supplementary materials:  crystallographic information; 3D view; checkCIF report

## Figures and Tables

**Fig. 1.**
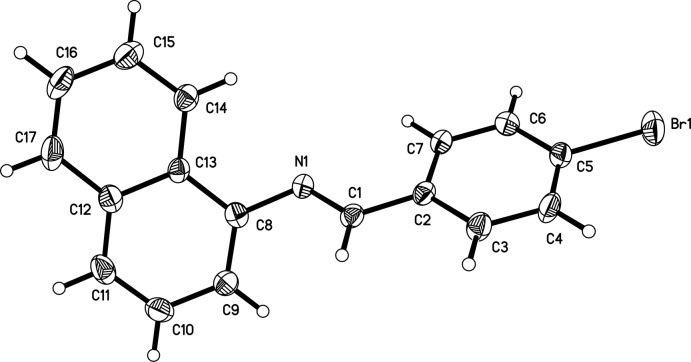
The molecular structure of the title compound with displacement ellipsoids drawn at the 30% probability level.

## supplementary crystallographic information

### Comment

Schiff bases have been receiving considerable attention for many years, primarily due to their importance as ligands in metal complexes with special magnetic (Weber *et al.*, 2007), catalytic (Chen *et al.*, 2008) and biological properties (May *et al.*,2004). As a part of our studies on the synthesis and structural properties of Schiff bases with naphthylamine and arylaldehydes, we have determined the structure of the title compound (Fig. 1). The molecule is in a *trans* configuration with respect to the C11═N1 bond. The mean planes of the naphthylene ring system and benzene ring, C1—C10 and C12—C17 respectively, form dihedral angle of 53.26 (3)°. Some examples of related structures already appear in the literature (Zhu *et al.*, 2010; Harada *et al.*, 2004; Tariq *et al.*, 2010).

### Experimental

1-Naphthylamine (0.72 g,5 mmol) and *p*-bromobenzaldehyde (0.92 g, 5 mmol) were dissolved in ethanol (30 ml). The mixture was refluxed for 2 h, and then cooled to room temperature. The reaction mixture was filtered and the filter cake was recreystallized from ethyl alcohol (yield 90%). Crystals suitable for X-ray diffraction were obtained by slow evaporation of an ethanol solution of the title compound.

### Refinement

H atoms were placed in idealized positions and allowed to ride on their respective parent atoms, with C—H = 0.93 Å and *U*_iso_(H) = 1.2*U*_eq_(C).

### Crystal data

**Table:**

C_17_H_12_BrN	*F*(000) = 624
*M**_r_* = 310.19	*D*_x_ = 1.508 Mg m^−^^3^
Monoclinic, *P*2_1_/*c*	Mo *K*α radiation, λ = 0.71073 Å
Hall symbol: -P 2ybc	Cell parameters from 2253 reflections
*a* = 7.0823 (6) Å	θ = 2.7–24.1°
*b* = 25.555 (2) Å	µ = 2.99 mm^−^^1^
*c* = 7.5712 (5) Å	*T* = 298 K
β = 94.431 (1)°	Block, yellow
*V* = 1366.19 (18) Å^3^	0.45 × 0.41 × 0.28 mm
*Z* = 4	

### Data collection

**Table:**

Bruker SMART CCD diffractometer	2405 independent reflections
Radiation source: fine-focus sealed tube	1643 reflections with *I* > 2σ(*I*)
Graphite monochromator	*R*_int_ = 0.080
φ and ω scans	θ_max_ = 25.0°, θ_min_ = 2.8°
Absorption correction: multi-scan (*SADABS*; Sheldrick, 1996)	*h* = −8→8
*T*_min_ = 0.346, *T*_max_ = 0.488	*k* = −24→30
6750 measured reflections	*l* = −9→8

### Refinement

**Table:**

Refinement on *F*^2^	Secondary atom site location: difference Fourier map
Least-squares matrix: full	Hydrogen site location: inferred from neighbouring sites
*R*[*F*^2^ > 2σ(*F*^2^)] = 0.043	H-atom parameters constrained
*wR*(*F*^2^) = 0.100	*w* = 1/[σ^2^(*F*_o_^2^) + (0.0289*P*)^2^ + 0.2762*P*] where *P* = (*F*_o_^2^ + 2*F*_c_^2^)/3
*S* = 1.03	(Δ/σ)_max_ = 0.001
2405 reflections	Δρ_max_ = 0.45 e Å^−^^3^
173 parameters	Δρ_min_ = −0.32 e Å^−^^3^
0 restraints	Extinction correction: *SHELXL97* (Sheldrick, 2008), Fc^*^=kFc[1+0.001xFc^2^λ^3^/sin(2θ)]^-1/4^
Primary atom site location: structure-invariant direct methods	Extinction coefficient: 0.0366 (19)

### Special details

**Table:**

Geometry. All e.s.d.'s (except the e.s.d. in the dihedral angle between two l.s. planes) are estimated using the full covariance matrix. The cell e.s.d.'s are taken into account individually in the estimation of e.s.d.'s in distances, angles and torsion angles; correlations between e.s.d.'s in cell parameters are only used when they are defined by crystal symmetry. An approximate (isotropic) treatment of cell e.s.d.'s is used for estimating e.s.d.'s involving l.s. planes.
Refinement. Refinement of *F*^2^ against ALL reflections. The weighted *R*-factor *wR* and goodness of fit *S* are based on *F*^2^, conventional *R*-factors *R* are based on *F*, with *F* set to zero for negative *F*^2^. The threshold expression of *F*^2^ > σ(*F*^2^) is used only for calculating *R*-factors(gt) *etc*. and is not relevant to the choice of reflections for refinement. *R*-factors based on *F*^2^ are statistically about twice as large as those based on *F*, and *R*- factors based on ALL data will be even larger.

### Fractional atomic coordinates and isotropic or equivalent isotropic displacement parameters (Å^2^)

**Table:**

	*x*	*y*	*z*	*U*_iso_*/*U*_eq_	
Br1	−0.13531 (5)	0.479652 (16)	0.74533 (7)	0.0722 (3)	
N1	0.4392 (3)	0.26397 (10)	0.6574 (4)	0.0388 (7)	
C1	0.4699 (4)	0.30922 (13)	0.7241 (4)	0.0368 (8)	
H1	0.5918	0.3173	0.7707	0.044*	
C2	0.3235 (4)	0.34896 (12)	0.7310 (4)	0.0353 (8)	
C3	0.3651 (5)	0.39638 (13)	0.8152 (5)	0.0452 (9)	
H3	0.4857	0.4018	0.8698	0.054*	
C4	0.2310 (5)	0.43560 (13)	0.8194 (5)	0.0497 (10)	
H4	0.2610	0.4674	0.8744	0.060*	
C5	0.0531 (5)	0.42671 (13)	0.7408 (5)	0.0429 (9)	
C6	0.0059 (5)	0.38013 (15)	0.6560 (5)	0.0492 (10)	
H6	−0.1149	0.3749	0.6016	0.059*	
C7	0.1413 (4)	0.34164 (13)	0.6537 (5)	0.0475 (10)	
H7	0.1100	0.3099	0.5989	0.057*	
C8	0.5943 (4)	0.22972 (13)	0.6450 (4)	0.0371 (8)	
C9	0.7594 (4)	0.24571 (14)	0.5768 (5)	0.0453 (10)	
H9	0.7726	0.2804	0.5422	0.054*	
C10	0.9082 (5)	0.21025 (16)	0.5588 (5)	0.0546 (11)	
H10	1.0187	0.2217	0.5121	0.066*	
C11	0.8930 (5)	0.15948 (15)	0.6086 (5)	0.0558 (11)	
H11	0.9932	0.1366	0.5960	0.067*	
C12	0.7270 (5)	0.14119 (14)	0.6791 (5)	0.0440 (10)	
C13	0.5735 (4)	0.17643 (12)	0.6966 (4)	0.0343 (8)	
C14	0.4062 (5)	0.15755 (13)	0.7641 (4)	0.0430 (9)	
H14	0.3048	0.1801	0.7752	0.052*	
C15	0.3920 (5)	0.10638 (15)	0.8134 (5)	0.0552 (10)	
H15	0.2805	0.0945	0.8570	0.066*	
C16	0.5417 (6)	0.07172 (15)	0.7995 (6)	0.0609 (12)	
H16	0.5307	0.0371	0.8351	0.073*	
C17	0.7043 (6)	0.08871 (15)	0.7337 (6)	0.0585 (11)	
H17	0.8033	0.0652	0.7242	0.070*	

### Atomic displacement parameters (Å^2^)

**Table:**

	*U*^11^	*U*^22^	*U*^33^	*U*^12^	*U*^13^	*U*^23^
Br1	0.0609 (3)	0.0474 (3)	0.1084 (5)	0.01858 (19)	0.0070 (3)	−0.0096 (3)
N1	0.0399 (15)	0.0300 (16)	0.0466 (19)	0.0018 (12)	0.0048 (13)	−0.0012 (14)
C1	0.0397 (18)	0.033 (2)	0.038 (2)	−0.0005 (15)	0.0021 (15)	0.0049 (16)
C2	0.0434 (19)	0.0287 (19)	0.034 (2)	−0.0012 (15)	0.0055 (16)	0.0028 (15)
C3	0.047 (2)	0.036 (2)	0.051 (2)	0.0023 (16)	−0.0061 (18)	0.0006 (18)
C4	0.059 (2)	0.0260 (19)	0.063 (3)	0.0008 (17)	−0.001 (2)	−0.0061 (18)
C5	0.0459 (19)	0.032 (2)	0.052 (2)	0.0059 (16)	0.0117 (17)	0.0064 (18)
C6	0.0381 (19)	0.042 (2)	0.068 (3)	−0.0009 (17)	0.0076 (18)	−0.0029 (19)
C7	0.044 (2)	0.029 (2)	0.070 (3)	−0.0028 (16)	0.0113 (18)	−0.0076 (19)
C8	0.0351 (17)	0.040 (2)	0.035 (2)	0.0026 (15)	−0.0031 (15)	−0.0061 (16)
C9	0.0435 (19)	0.038 (2)	0.054 (3)	−0.0024 (16)	0.0033 (18)	−0.0066 (18)
C10	0.0395 (19)	0.053 (3)	0.073 (3)	−0.0048 (18)	0.0131 (19)	−0.015 (2)
C11	0.040 (2)	0.046 (3)	0.080 (3)	0.0071 (17)	−0.004 (2)	−0.024 (2)
C12	0.0397 (19)	0.038 (2)	0.053 (2)	0.0018 (16)	−0.0073 (18)	−0.0130 (18)
C13	0.0382 (17)	0.0290 (19)	0.035 (2)	0.0007 (15)	−0.0022 (15)	−0.0066 (16)
C14	0.050 (2)	0.035 (2)	0.044 (2)	0.0000 (16)	0.0041 (17)	−0.0072 (18)
C15	0.070 (3)	0.041 (2)	0.055 (3)	−0.009 (2)	0.014 (2)	−0.001 (2)
C16	0.078 (3)	0.030 (2)	0.073 (3)	−0.006 (2)	−0.005 (2)	0.006 (2)
C17	0.063 (2)	0.034 (2)	0.076 (3)	0.0115 (19)	−0.013 (2)	−0.008 (2)

### Geometric parameters (Å, º)

**Table:**

Br1—C5	1.902 (3)	C9—C10	1.405 (5)
N1—C1	1.274 (4)	C9—H9	0.9300
N1—C8	1.413 (4)	C10—C11	1.358 (5)
C1—C2	1.455 (4)	C10—H10	0.9300
C1—H1	0.9300	C11—C12	1.408 (5)
C2—C7	1.388 (4)	C11—H11	0.9300
C2—C3	1.390 (5)	C12—C17	1.416 (5)
C3—C4	1.383 (5)	C12—C13	1.426 (5)
C3—H3	0.9300	C13—C14	1.412 (4)
C4—C5	1.370 (5)	C14—C15	1.366 (5)
C4—H4	0.9300	C14—H14	0.9300
C5—C6	1.381 (5)	C15—C16	1.392 (5)
C6—C7	1.375 (5)	C15—H15	0.9300
C6—H6	0.9300	C16—C17	1.361 (6)
C7—H7	0.9300	C16—H16	0.9300
C8—C9	1.376 (5)	C17—H17	0.9300
C8—C13	1.427 (4)		
			
C1—N1—C8	118.6 (3)	C8—C9—H9	119.7
N1—C1—C2	123.2 (3)	C10—C9—H9	119.7
N1—C1—H1	118.4	C11—C10—C9	120.9 (4)
C2—C1—H1	118.4	C11—C10—H10	119.6
C7—C2—C3	117.9 (3)	C9—C10—H10	119.6
C7—C2—C1	122.2 (3)	C10—C11—C12	120.6 (3)
C3—C2—C1	119.9 (3)	C10—C11—H11	119.7
C4—C3—C2	121.4 (3)	C12—C11—H11	119.7
C4—C3—H3	119.3	C11—C12—C17	122.8 (3)
C2—C3—H3	119.3	C11—C12—C13	119.4 (3)
C5—C4—C3	118.7 (3)	C17—C12—C13	117.8 (3)
C5—C4—H4	120.6	C14—C13—C12	119.0 (3)
C3—C4—H4	120.6	C14—C13—C8	122.2 (3)
C4—C5—C6	121.7 (3)	C12—C13—C8	118.8 (3)
C4—C5—Br1	119.8 (3)	C15—C14—C13	120.5 (3)
C6—C5—Br1	118.5 (2)	C15—C14—H14	119.7
C7—C6—C5	118.6 (3)	C13—C14—H14	119.7
C7—C6—H6	120.7	C14—C15—C16	121.1 (4)
C5—C6—H6	120.7	C14—C15—H15	119.5
C6—C7—C2	121.7 (3)	C16—C15—H15	119.5
C6—C7—H7	119.2	C17—C16—C15	119.7 (4)
C2—C7—H7	119.2	C17—C16—H16	120.1
C9—C8—N1	121.9 (3)	C15—C16—H16	120.1
C9—C8—C13	119.6 (3)	C16—C17—C12	121.9 (4)
N1—C8—C13	118.4 (3)	C16—C17—H17	119.1
C8—C9—C10	120.7 (3)	C12—C17—H17	119.1
			
C8—N1—C1—C2	175.2 (3)	C9—C10—C11—C12	0.2 (6)
N1—C1—C2—C7	−4.6 (5)	C10—C11—C12—C17	−179.7 (3)
N1—C1—C2—C3	176.3 (3)	C10—C11—C12—C13	0.5 (5)
C7—C2—C3—C4	−1.2 (6)	C11—C12—C13—C14	178.8 (3)
C1—C2—C3—C4	178.0 (3)	C17—C12—C13—C14	−1.0 (5)
C2—C3—C4—C5	1.0 (6)	C11—C12—C13—C8	−1.2 (5)
C3—C4—C5—C6	−1.0 (6)	C17—C12—C13—C8	179.0 (3)
C3—C4—C5—Br1	179.6 (3)	C9—C8—C13—C14	−178.7 (3)
C4—C5—C6—C7	1.1 (6)	N1—C8—C13—C14	−2.0 (5)
Br1—C5—C6—C7	−179.5 (3)	C9—C8—C13—C12	1.3 (5)
C5—C6—C7—C2	−1.3 (6)	N1—C8—C13—C12	178.0 (3)
C3—C2—C7—C6	1.3 (6)	C12—C13—C14—C15	0.5 (5)
C1—C2—C7—C6	−177.8 (4)	C8—C13—C14—C15	−179.5 (3)
C1—N1—C8—C9	−48.1 (5)	C13—C14—C15—C16	0.4 (6)
C1—N1—C8—C13	135.2 (3)	C14—C15—C16—C17	−0.9 (6)
N1—C8—C9—C10	−177.2 (3)	C15—C16—C17—C12	0.4 (6)
C13—C8—C9—C10	−0.6 (5)	C11—C12—C17—C16	−179.2 (4)
C8—C9—C10—C11	−0.1 (6)	C13—C12—C17—C16	0.5 (6)
